# Analgesic interventions in surgically castrated beef calves: impacts on biomarkers, thermography, and growth performance under tropical conditions

**DOI:** 10.1007/s11250-025-04832-7

**Published:** 2026-02-02

**Authors:** Brahian Camilo Tuberquia-López, María Juliana Loaiza-Escobar, Marcela Patricia Eraso-Cadena, Jorge Guillermo Noriega Márquez, Nathalia M. Correa-Valencia

**Affiliations:** 1 GINVER, Facultad de Medicina Veterinaria, Corporación Universitaria Remington, calle 51 n.° 51-27, Medellín, Colombia; 2 Corporación Colombiana de Investigación Agropecuaria - AGROSAVIA, Centro de Investigación El Nus., San Roque - Antioquia, Colombia; 3 CENTAURO, Escuela de Medicina Veterinaria, Facultad de Ciencias Agrarias, Universidad de Antioquia UdeA, Calle 70 No. 52-21, Medellín, Colombia

**Keywords:** Analgesic protocols, Animal welfare, Beef cattle, Blanco orejinegro, Castration, Cortisol, Haptoglobin, Infrared thermography

## Abstract

Castration is common in beef cattle for reproductive and management purposes, but it causes pain and stress, raising welfare concerns. The adoption of perioperative anesthesia and analgesia remains uneven in tropical low- and middle-income settings, where infrastructure, costs, and weak enforcement limit its application. This study aimed to evaluate the effects of different analgesic protocols on physiological stress markers and growth performance in beef calves raised under tropical conditions in Colombia. Forty-two intact Blanco Orejinegro (BON) calves were randomly assigned to three protocols: spermatic cord block with lidocaine plus tolfenamic acid (T1), low epidural anesthesia with lidocaine plus tolfenamic acid (T2), or tolfenamic acid alone (T3). All calves underwent closed surgical castration. Physiological and welfare indicators were assessed through pulse rate variability (PRV), hematological parameters, serum haptoglobin and cortisol concentrations, and infrared thermography (IRT) of the scrotal area at defined intervals, whereas growth performance was evaluated by average daily gain (ADG). Statistical analyses included ANOVA and nonparametric tests when assumptions were violated, with multiple comparisons corrected via Tukey’s test or Dunn’s test. No significant differences were found among the protocols for the PRV, hematological parameters, cortisol, haptoglobin, IRT, or ADG. In contrast, significant temporal effects were observed, with cortisol decreasing at 48 h, haptoglobin peaking at 48–72 h, and IRT values increasing between 24 and 48 h postcastration. These results indicate that the time after castration, rather than the analgesic protocol, was the primary driver of physiological responses.

**Supplementary Information:**

The online version contains supplementary material available at 10.1007/s11250-025-04832-7.

## Introduction

Castration is a widespread management practice in beef cattle production systems worldwide and is performed primarily to prevent unwanted reproduction, improve temperament, and enhance carcass quality (Stafford and Mellor 2005). Despite these benefits, it remains a contentious issue due to animal welfare concerns. While producers often regard castration as a routine and necessary procedure, the public and meat consumer increasingly perceive it as unnecessarily painful (Coetzee 2011).

All commonly employed castration methods, including surgical removal of the testes and nonsurgical approaches such as rubber rings, latex bands, or the Burdizzo clamp, induce physiological, neuroendocrine, and behavioral responses indicative of pain and distress (Marti et al. 2017; Meléndez et al. 2017). Castration commonly causes transient *growth checks* due to pain, inflammation, and reduced feed intake; the penalty is generally greater when castration occurs at older ages, whereas earlier castration and effective analgesia reduce short-term performance losses (Bretschneider 2005; Marquette et al. 2020). Meta-analyses and controlled trials have shown that multimodal pain management mitigates acute physiological stress and can improve early postprocedure average daily gain and some carcass traits, supporting its routine use in beef systems (Nogues et al. 2025). This can be alleviated via the use of local or epidural anesthesia combined with systemic analgesics such as nonsteroidal anti-inflammatory drugs (NSAIDs) (Ting et al. 2003; Johnstone et al. 2021). Nevertheless, in many low- and middle-income countries, including Colombia, castration is frequently performed without any pain mitigation due to limited veterinary infrastructure, costs, and lack of regulatory enforcement (Tuberquia-López et al. 2024; del Campo et al. 2025). In contrast, producers in developed countries are increasingly adopting analgesic protocols, reflecting both evolving welfare standards and consumer expectations. For example, in high-income settings, castration protocols increasingly embed analgesia—typically local lidocaine plus an NSAID—and several jurisdictions require or direct its use (e.g., Canada > 6 months; New Zealand/Australia beyond early ages) (Animal Health Australia 2016; New Zealand Government 2018; National Farm Animal Care Council 2023). Professional bodies—notably the Federation of Veterinarians of Europe (FVE, 2020)—emphasize that anesthesia/analgesia is a veterinary act, underscoring preprocedure assessment and monitoring. Together, these policies offer a practical model of pain control integrated into the castration procedure.

Castration elicits measurable changes in multiple biomarkers of stress and inflammation. These include elevated plasma and salivary cortisol, increased substance P concentrations—a neuropeptide associated with nociception—and heightened levels of haptoglobin, an acute-phase protein that typically peaks 2–3 days after castration (Earley and Crowe 2002; Tschoner and Feist 2022). These biomarkers provide objective tools for evaluating pain and assessing the effectiveness of analgesic interventions. In addition to traditional physiological markers, noninvasive approaches, such as pulse rate variability (PRV), which reflects autonomic nervous system balance, and infrared thermography (IRT), which detects stress-related changes in peripheral blood flow, have emerged as promising complementary indicators of animal discomfort and welfare status (Stewart et al. 2005, 2010). Noninvasive metrics such as the PRV and IRT are rapid, repeatable, and low-stress, enabling on-farm, repeated measurements with minimal restraint (Kovács et al. 2014; Mota-Rojas et al. 2002). The PRV sensitively tracks autonomic balance (sympathetic–parasympathetic) in real time, whereas IRT maps superficial temperature to detect localized inflammation/perfusion changes—often earlier than overt clinical signs (Stewart et al. 2010).

In addition to traditional physiological markers, noninvasive approaches such as pulse rate variability (PRV) and infrared thermography (IRT) offer practical advantages: they are rapid, repeatable, and low-stress/low-contact for cattle, enabling on-farm, repeated measurements. PRV indices autonomic balance (sympathetic–parasympathetic) with high temporal resolution, whereas IRT visualizes localized heat patterns linked to inflammation and altered perfusion, helping detect pain-related changes earlier than clinical signs.

Colombia maintained approximately 29.4 million cattle during the second foot-and-mouth disease (FMD) vaccination cycle of 2024 (FEDEGAN, 2025). Within this total, Antioquia is the leading department, concentrating ~ 11% of the national herd (> 3 million head) and encompassing mixed beef, dual-purpose, and specialized dairy systems across tropical and high-tropical zones.

Given the limited evidence from tropical environments and the scarcity of enforceable welfare policies in countries such as Colombia, the benefits of pain-management protocols during castration remain underexplored. Accordingly, we hypothesized that multimodal analgesia at castration would reduce acute physiological stress responses and enhance growth performance relative to less comprehensive pain control.

## Materials and methods

### Ethical considerations

The study was conducted in accordance with national and institutional guidelines for the care and use of animals in research. The experimental protocol was reviewed and approved by the Bioethics Committee for Research with Animals (CIBA) of the Faculty of Veterinary Medicine, Corporación Universitaria Remington (Acta 05-2021, Colombia).

### Calves and housing

A total of 42 intact male preweaned cattle aged 5–6 months of the Colombian native Blanco Orejinegro breed (BON) were included in the experiment. The animals were managed in a beef cattle herd under lowland tropical conditions in Northeast Antioquia (Colombia) at an altitude of 800 m.a.s.l., with a mean annual temperature of 23 °C. All calves were maintained on continuous grazing pastures, with unrestricted access to water and supplementation with mineralized salt.

### Study design

One week prior to the study, calves were moved to the handling pens to allow acclimatization to management routines and minimize stress during experimental procedures. The animals were randomly allocated into three treatment groups (*n* = 14 per group):

- *Treatment 1 (T1)*: A sperm cord block with 10 mL of 2% lidocaine (Lidocaine 2%^®^, Corpaul, Medellín, Colombia) was administered per cord, combined with tolfenamic acid (Tolfen LA^®^, Lima, Peru) at 4 mg/kg IM at a single preoperative dose.
- *Treatment 2 (T2)*: Low epidural anesthesia using 4 mL of 2% lidocaine plus tolfenamic acid (Tolfen LA^®^, Lima, Peru) at 4 mg/kg IM in a single preoperative dose.
- *Treatment 3 (T3)*: Tolfenamic acid (Tolfen LA^®^, Lima, Peru) at 4 mg/kg IM was administered once before the procedure.

For all the treatments, the scrotal region was surgically prepared, and closed castration was performed via an emasculator following the technique described by Hendrickson and Baird (2013). Animals were restrained in a chute, sedated with intravenous xylazine at 0.02 mg/kg (Rompun^®^, Bayer Animal Health, Leverkusen, Germany) prior to castration, and received benzathine penicillin at 11,000 IU/kg IM (Benzetacil LA^®^, Elanco Animal Health, Greenfield, IN, USA) in a single dose at the end of the procedure as prophylaxis. All anesthesia/analgesia administrations and surgical castrations were performed by an experienced veterinarian specializing in the procedure (BT).

### Pulse rate, hematology, biomarkers, thermography, and growth performance

The pulse rate was continuously recorded during castration with a portable oximeter (CMS 60D, Contec Medical Systems, Qinhuangdao, China). With calves restrained in a chute, the probe was placed on the preputial mucosa to ensure a stable signal. Recording began immediately before castration and continued at 30-s intervals until the procedure was completed. The duration of castration ranged from 3 to 6 min, yielding approximately 6–12 pulse rate readings per calf.

Hematological variables (RBC, Hb, HCT, WBC with neutrophils and lymphocytes, and platelets) were measured at 0 and 24 h via an automated veterinary analyzer (Mindray BC-2800 Vet^®^, Mindray Biomedical Electronics, Shenzhen, China). Differential leukocyte counts were obtained from Wright-stained smears examined under oil immersion at 100×.

Serum haptoglobin was quantified at 0, 24, 48, and 72 h with a bovine ELISA kit (Abbexa^®^, Cambridge, UK; Cat. No. abx258067). Precision was confirmed by intra- and interassay CVs (< 10% and < 12%, respectively). A standard curve (15.6–1000 ng/mL) was prepared, the absorbance was read at 450 nm with a microplate reader (Thermo Fisher Multiskan^®^ FC, Waltham, MA, USA), and the concentrations were interpolated via a four-parameter logistic regression model.

Serum cortisol was determined at 0, 24, 48, and 72 h via a commercial ELISA kit (AccuBind^®^ Cortisol Assay, Monobind Inc., Lake Forest, CA, USA; sensitivity 91.5 pg). The optical density was read at 450 nm with a spectrophotometer (BioTek^®^ ELx800, Winooski, VT, USA). A 0–20 µg/dL standard curve was prepared in duplicate, and concentrations were calculated via four-parameter logistic regression in R (v4.5.1, drc package), with a model fit R² = 0.9993 (RMSE = 0.1887).

Infrared thermographic images of scrotal wounds were obtained at 24, 48, and 72 h with a FLIR ONE Pro thermal camera (FLIR Systems Inc., Wilsonville, OR, USA) connected to a smartphone. Images were taken at ~ 50 cm in shaded conditions with calves calmly restrained. The emissivity was set at 0.98, and the ambient temperature and humidity were recorded. The data were processed with FLIR Tools software to analyze the wound temperature gradients.

Body weight was measured 15 d before castration and 30 d after castration with a calibrated electronic livestock scale, zeroed and verified with test weights before each session. The calves were weighed individually after a brief stabilization period. The average daily gain (ADG) was calculated as (BWfinal – BWinitial)/Δt, where Δt was the number of days between weighings.

### Statistical analysis

Each calf was considered an experimental unit. The effects of analgesic interventions on inflammatory biomarkers, the PRV, IRT, and ADG were analyzed. For continuous outcomes with a normal distribution and homogeneous variances, analysis of variance (ANOVA) was applied, with post hoc Tukey’s test for pairwise comparisons. Data that violated normality (Shapiro–Wilk test) or homogeneity of variance (Levene’s test) were analyzed via nonparametric tests: Kruskal–Wallis for between-group comparisons and Friedman’s test for repeated measures, followed by Dunn’s post hoc tests with Bonferroni correction. The results are presented as the means ± standard deviations (SDs) or medians [interquartile ranges (IQRs)], as appropriate. All analyses were conducted via R software (v. 4.5.0; R Foundation for Statistical Computing, Vienna, Austria).

## Results

### Pulse rate monitoring

PRV was monitored continuously during castration across the three treatment groups, with similar median values: 59 (49.75–71) bpm for T1, 60 (49–86) bpm for T2, and 61 (56–69) bpm for T3 (table and figure in supplementary material 1). Therefore, a Kruskal–Wallis’s test was applied, revealing no significant differences among the treatments (*p* = 0.563).

### Hematological profile

Hematological parameters were evaluated at 0 and 24 h postcastration. No significant intergroup differences were detected in the following parameters: leukocytes (*p* = 0.115), lymphocytes (*p* = 0.312), neutrophils (*p* = 0.166), red blood cells (RBCs) (*p* = 0.198), hemoglobin (*p* = 0.126), hematocrit (*p* = 0.131), or platelets (*p* = 0.307). Overall, the hematological profiles remained stable across treatments during the first 24 h, with no relevant changes in red cell indices or leukocyte counts (the complete values are presented in supplementary material 1).

### Haptoglobin and cortisol analysis

The median haptoglobin concentrations increased after castration in all the treatments, peaking at 48 h and remaining elevated at 72 h. The baseline values were similar (128–138 mg/dL), but by 48 h, they had increased to 269.3 mg/dL in T1, 216.8 mg/dL in T2, and 213.5 mg/dL in T3 (Table 1). At 72 h, T3 maintained the highest concentration (316.8 mg/dL), whereas T1 declined slightly and T2 remained stable (figure as supplementary material 1). No treatment effect was detected (χ² = 0.33, *p* = 0.849), but significant temporal changes were observed (χ² = 24.56, *p* < 0.001). Pairwise tests confirmed significant increases from 0 h to 48 h (*p* = 0.0014) and 72 h (*p* = 0.0126) and from 24 h to both 48 h (*p* = 0.0021) and 72 h (*p* = 0.0198), whereas no differences were found between 0 and 24 h or between 48 and 72 h.

Serum cortisol concentrations varied primarily as a function of time after castration, with no significant differences among treatments (Kruskal–Wallis, *p* = 0.675). In contrast, both the Kruskal–Wallis test (*p* = 0.004) and the Friedman test (*p* = 0.007) revealed significant temporal effects.

Specifically, the cortisol level decreased significantly from baseline (0 h) to 48 h postcastration (*p* = 0.002), with partial recovery by 72 h, although it did not reach the initial value (Table 1). The distribution of data indicated marked interindividual variability, especially among calves receiving T1, where values ranged widely within the first 24 h. In contrast, calves in the T2 and T3 groups exhibited more homogeneous patterns, with median concentrations remaining comparatively stable (figure as supplementary material 1). A summary of the inferential statistics for haptoglobin and cortisol is presented in Table 2.

**Table 1:** Serum haptoglobin and cortisol concentrations in beef calves cast under three analgesic protocols in tropical Colombia

Treatment	Overall median	0 h	24 h	48 h	72 h
**Serum haptoglobin (ng/mL)**					
T1	175.68 [110–246.73]	138.07 [81.85–176.32]	165.13 [130.03–182.29]	269.31 [202.61–349.98]	168.83 [9.4–315.77]
T2	151.98 [109.48–225.03]	132.13 [109.09–155.27]	144.06 [111.34–162.75]	216.82 [148.16–285.62]	213.08 [90.28–320.78]
T3	159.49 [110.78–253.76]	127.95 [106.89–159.79]	131.85 [112.62–150.06]	213.51 [163.36–271.01]	316.76 [177.49–353.94]
**Serum cortisol (µg/dL)**					
T1	2.34 [1.29–1.06]	3.51 [2.34–4.89]	2.89 [1.14–6.29)	1.54 [1.27–2.18)	2.28 [1.51–2.85]
T2	2.29 [1.65–3.73]	2.58 [1.88–3.65]	1.9 [1.43–2.82]	2.15 [1.69–3.05]	2.54 [1.85–4.18]
T3	2.03 [1.54–3.13]	2.87 [2.17–4.22]	2.41 [1.75–3.40]	1.6 [1.49–2.17]	1.71 [1.41–2.52]

T1 = Spermatic cord block with lidocaine (10 mL, 2% per cord) + tolfenamic acid (4 mg/kg IM, preop); T2 = Low epidural anesthesia with lidocaine (4 mL, 2%) + tolfenamic acid (4 mg/kg IM, preop); T3 = Tolfenamic acid only (4 mg/kg IM, preop)

**Table 2:** Summary of statistical tests for haptoglobin and cortisol (global and pairwise)

Outcome	Effect tested	Test/statistic	*p* value	
Haptoglobin	Treatment effect (T1 vs. T2 vs. T3)	χ² = 0.33	0.849	
Haptoglobin	Time effect (0, 24, 48, 72 h)	χ² = 24.56	< 0.001	
Haptoglobin	Pairwise (time)	0 h vs. 48 h	0.0014	
		0 h vs. 72 h	0.0126	
		24 h vs. 48 h	0.0021	
		24 h vs. 72 h	0.0198	
		0 h vs. 24 h	0. ns	
		48 h vs. 72 h	0. ns	
Cortisol	Treatment effect (T1 vs. T2 vs. T3)	Kruskal–Wallis	0.675	
Cortisol	Time effect	Kruskal–Wallis	0.004	
Cortisol	Time effect	Friedman	0.007	
Cortisol	Pairwise (time)	0 h vs. 48 h	0.002	

T1 = Spermatic cord block with lidocaine (10 mL, 2% per cord) + tolfenamic acid (4 mg/kg IM, preop); T2 = Low epidural anesthesia with lidocaine (4 mL, 2%) + tolfenamic acid (4 mg/kg IM, preop); T3 = Tolfenamic acid only (4 mg/kg IM, preop)

### IRT

Scrotal surface temperature, as measured by IRT, significantly varied over time after castration, whereas treatment effects were less pronounced. The Kruskal–Wallis test revealed no clear differences among the treatments (*p* = 0.054), but both the time (*p* < 0.001) and the Friedman test confirmed significant temporal effects (*p* < 0.001). Overall, calves in the T3 group presented slightly higher median temperatures (33.7 °C) than those in the T1 (32.9 °C) and T2 (33.1 °C) groups did, although these differences were not statistically significant (Table 3).

**Table 3:** Summary of scrotal surface temperature (°C) in beef calves cast under three analgesic protocols in tropical Colombia

Treatment	Description	*n*	Median	Q1	Q3	IQR
T1	Spermatic cord block with lidocaine (10 mL, 2% per cord) + tolfenamic acid (4 mg/kg IM, preop)	42	32.9	30.40	33.60	3.20
T2	Low epidural anesthesia with lidocaine (4 mL, 2%) + tolfenamic acid (4 mg/kg IM, preop)	42	33.1	31.50	34.00	2.50
T3	Tolfenamic acid only (4 mg/kg IM, preop)	42	33.7	32.18	34.85	2.67

The values are expressed as medians, first quartiles (Q1), third quartiles (Q3), and interquartile ranges (IQRs)

The median temperatures increased from 24 h to 48 h postcastration, with values stabilizing at 72 h (table and figure as supplementary material 1). Pairwise comparisons revealed that scrotal temperatures at 24 h were significantly lower than those at 48 h (*p* < 0.01) and 72 h (*p* < 0.01), whereas no significant differences were detected between 48 and 72 h.

**Fig. 1:**
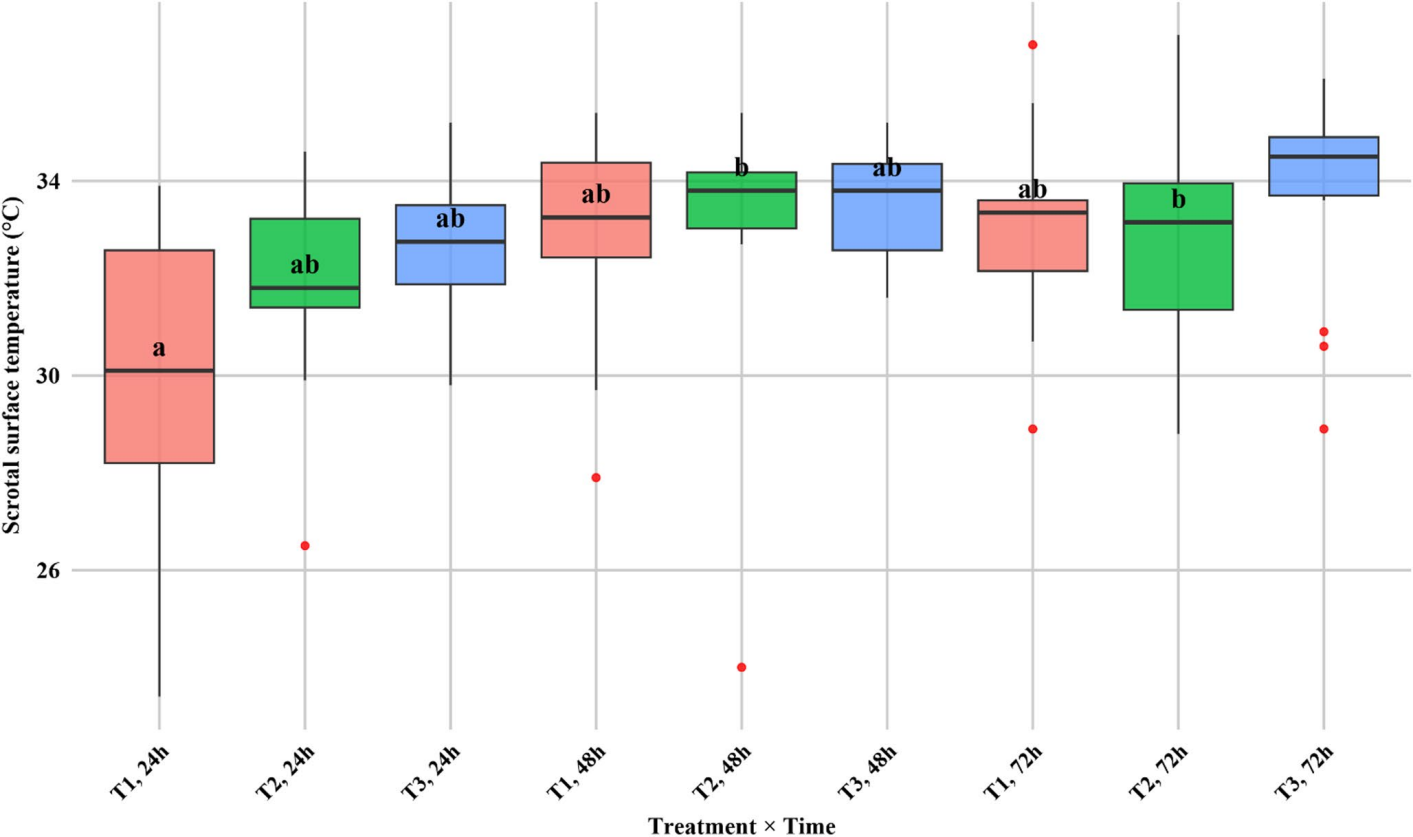
Interaction between treatment and time on scrotal surface temperature (°C) in beef calves cast under three analgesic protocols in tropical Colombia. Boxplots represent medians, interquartile ranges, and outliers. Different letters indicate significant differences between groups (*p* < 0.05)

At the treatment × time level, the T1 group presented the lowest scrotal temperature (median: 30.1 °C) at 24 h, which was significantly different from that at T2–48 h and T2–72 h (*p* < 0.05), indicating transient suppression of local inflammation in that subgroup (Fig. 1). Overall, IRT demonstrated sensitivity in detecting local inflammatory changes postcastration, with time after surgery being the main driver of variation, whereas treatment protocols exerted only subtle influences.

### ADG

Growth performance, expressed as ADG, did not differ significantly among the three analgesic protocols. The analysis of variance indicated no treatment effect (*p* = 0.269). Numerically, calves in the T1 group presented the highest mean ADG (1.31 ± 0.31 kg/day), followed by those in the T3 group (1.25 ± 0.20 kg/day), whereas those in the T2 group presented the lowest mean value (1.11 ± 0.40 kg/day). However, the wide standard deviations, particularly at T2, highlight the substantial variability among individuals. The median values were consistent with the means, ranging between 1.06 and 1.31 kg/day, and no treatment reached statistical superiority (table and figure in supplementary material 1).

## Discussion

Pain management during surgical castration remains a challenge in tropical systems, where field conditions often limit multimodal protocols (Steagall et al. 2021). In this study, three analgesic strategies were compared, but most physiological, inflammatory, and performance responses showed no treatment effect, suggesting that castration itself and recovery time were the main drivers of change (Coetzee 2011, 2013). Hematological variables, including RBC indices and leukocyte differentials, remained stable across groups during the first 24 h, which is consistent with reports that hematology is less sensitive to acute surgical stress than acute-phase proteins or endocrine markers are (Stafford and Mellor 2005; Cafazzo et al. 2012). This stability indicates that the inflammatory response is better reflected by biomarkers such as haptoglobin.

Haptoglobin concentrations rose significantly at 48 and 72 h after castration, which is consistent with the acute-phase response in cattle (Arthington et al. 2003). This increase was independent of treatment, indicating that tissue injury outweighed any modulatory effect of analgesics (Yoo et al. 2023). Similar delayed elevations have been reported in other bovine castration studies, confirming that haptoglobin is a reliable marker of inflammation and postoperative stress (Webster et al. 2013; Marti et al. 2017). In contrast, cortisol levels declined sharply at 48 h but partially recovered at 72 h, reflecting transient HPA axis activation (Fell et al. 1986). Although interindividual variability was noted—particularly at T1—no consistent treatment effects were detected, these findings support previous reports that cortisol responses are short-lived and often not fully mitigated by conventional analgesics (Ting et al. 2003; Petherick et al. 2014; Daniel et al. 2020).

IRT has a clear temporal pattern consistent with postcastration inflammation, where scrotal surface temperatures increase at 48 and 72 h (Stewart et al. 2010; Bergamasco et al. 2021), which aligns with the expected progression from early vascular changes to maximal hyperemia and edema as the local inflammatory cascade develops. The comparatively lower mean temperature at 24 h in the T1 group likely reflects the transient effect of the spermatic cord block—by attenuating nociception and sympathetic drive during and immediately after surgery, the block can blunt early vasodilatory responses and tissue perfusion changes, delaying heat expression at the skin (Stewart et al. 2005; Dockweiler et al. 2013; Polsky and von Keyserlingk 2017). By 48–72 h, as the block’s effect dissipates and leukocyte infiltration and fluid accumulation peak, surface temperature increases. Together with parallel changes in systemic biomarkers, this pattern supports IRT as a sensitive, noncontact indicator of local inflammation rather than a mere surrogate for ambient conditions (Bergamasco et al. 2021; Yamada et al. 2021). The average daily gain (ADG) did not differ among the treatments, although the variability and sample size may have limited the detection of subtle effects. Nevertheless, findings agree with previous reports that surgical castration has a modest short-term impact on growth when perioperative management is adequate (Webster et al. 2013; Roberts et al. 2018).

The present study provides relevant insights into the physiological, autonomic, and productive consequences of analgesic protocols for surgical castration under tropical beef production conditions. These findings highlight that effective pain management not only improves animal welfare indicators but also contributes to maintaining growth performance, reinforcing the link between welfare and productivity in sustainable livestock systems (Coetzee 2011; Stafford and Mellor 2011; Roberts et al. 2018).

Importantly, the study population consisted of BON cattle, a Colombian creole breed characterized by rusticity, adaptability to tropical highland environments, and resilience to nutritional and climatic stressors. Compared with specialized commercial breeds, BON cattle typically exhibit moderate growth rates, but they are valued for their efficiency in low-input systems, resistance to parasites, and high-quality carcass traits. These genetic and physiological characteristics may modulate stress and inflammatory responses to painful procedures, potentially influencing biomarker dynamics, thermoregulatory patterns, and growth outcomes observed in this study. Therefore, caution is warranted when extrapolating results to other breeds with different metabolic or adaptive profiles.

The study was conducted under field conditions, which, while enhancing external validity, limited the degree of experimental control. Sample size constraints in certain analyses may have reduced the statistical power to detect subtle effects of treatments. Additionally, the monitoring period was relatively short, with a focus on acute responses within the first 72 h and early growth trends, without capturing potential long-term consequences of analgesic protocols. Biomarker sampling was limited to 0–72 h to target the acute kinetics of cortisol and acute-phase proteins and to minimize repeated handling in a field setting; consequently, later trajectories were not captured, and future studies should extend sampling beyond 72 h and incorporate serial behavioral pain scoring. Finally, although multiple objective biomarkers have been used, integrating behavioral assessments could further strengthen welfare evaluations in future research.

In summary, the results of the castration of BON calves under tropical field conditions revealed that recovery time, rather than analgesic protocol, was the main determinant of physiological and growth responses. Haptoglobin and infrared thermography are reliable indicators of inflammation, whereas cortisol changes are transient, and hematology remains stable. None of the protocols impaired growth performance, and multimodal analgesia offered no clear benefit over tolfenamic acid alone. These findings highlight the practicality of NSAID use in field settings and the value of objective biomarkers for monitoring animal welfare.

## Electronic supplementary material

Below is the link to the electronic supplementary material.

Supplementary Material 1

## Data Availability

The data that support the findings of this study are available from the corresponding author upon reasonable request.
