# Supplementary material for: Analgesic interventions in surgically castrated beef calves: impacts on biomarkers, thermography, and growth performance under tropical conditions
Source: Trop Anim Health Prod. 2026 Feb 2;58(2):85. doi: 10.1007/s11250-025-04832-7 (PMC12864252; doi:10.1007/s11250-025-04832-7)
Supplement: Supplementary file 1 — Supplementary Material 1 [file 11250_2025_4832_MOESM1_ESM.docx]

**Table.** Pulse rate variability (PRV) in beef calves cast under three analgesic protocols in tropical Colombia.

| **Treatment** | **Description** | **n** | **Median (Q1–Q3) in bpm** |
| --- | --- | --- | --- |
| T1 | Spermatic cord block with lidocaine (10 mL, 2% per cord) + tolfenamic acid (4 mg/kg IM, preop) | 196 | 59 (49.75–71) |
| T2 | Low epidural anesthesia with lidocaine (4 mL, 2%) + tolfenamic acid (4 mg/kg IM, preop) | 196 | 60 (49–86) |
| T3 | Tolfenamic acid only (4 mg/kg IM, preop) | 196 | 61 (56–69) |

bpm = Beats per minute.

**
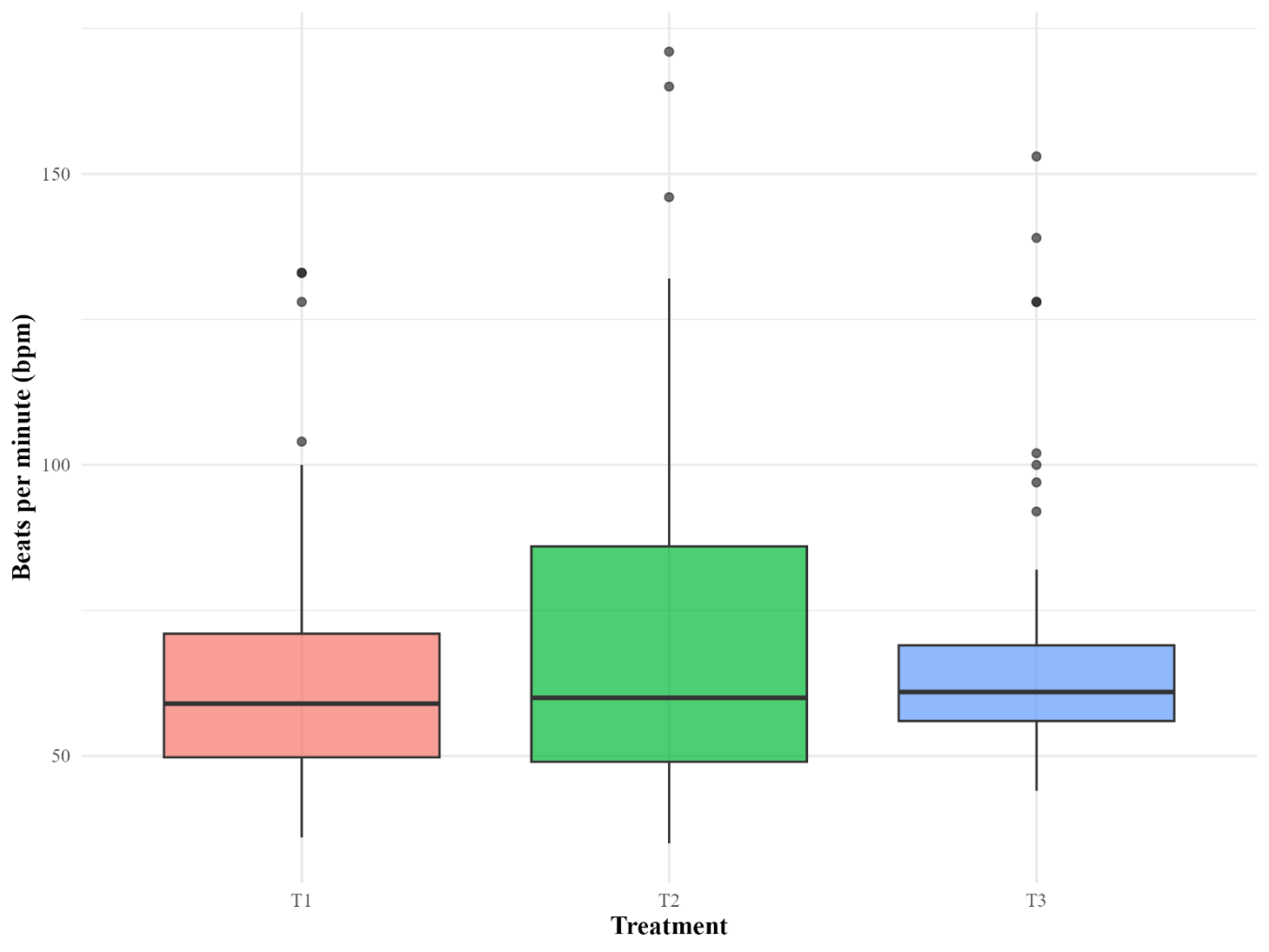
 Figure.** Distribution of pulse rate (in beats per minute—bpm) in beef calves cast under three analgesic protocols in tropical Colombia.

T1 = Spermatic cord block with lidocaine (10 mL, 2% per cord) + tolfenamic acid (4 mg/kg IM, preop); T2 = Low epidural anesthesia with lidocaine (4 mL, 2%) + tolfenamic acid (4 mg/kg IM, preop); T3 = Tolfenamic acid only (4 mg/kg IM, preop).

**Table.** Hematological parameters at baseline (0 h) and 24 h after castration in beef calves under three analgesic protocols in tropical Colombia.

| **Parameter** | **T1 (0 h)** | **T2 (0 h)** | **T3 (0 h)** | **T1 (24 h)** | **T2 (24 h)** | **T3 (24 h)** |
| --- | --- | --- | --- | --- | --- | --- |
| RBC (×10⁶/µL) | 8.7 | 8.6 | 8 | 8.2 | 7.8 | 7.7 |
| Hb (g/dL) | 10 | 10.3 | 9.2 | 9.6 | 9.3 | 9 |
| HCT (%) | 32.9 | 34.2 | 30.3 | 31.7 | 30.1 | 29.3 |
| WBC (×10³/µL) | 13.1 | 15.6 | 12.5 | 15.5 | 15.2 | 15 |
| Neutrophils (×10³/µL) | 4.1 | 5 | 3.8 | 5.9 | 6 | 5.1 |
| Lymphocytes (×10³/µL) | 7.7 | 9.1 | 7.5 | 8.4 | 8 | 8.6 |
| Platelets (×10³/µL) | 253.3 | 336 | 388 | 376.1 | 361.1 | 307 |

T1 = Spermatic cord block with lidocaine (10 mL, 2% per cord) + tolfenamic acid (4 mg/kg IM, preop); T2 = Low epidural anesthesia with lidocaine (4 mL, 2%) + tolfenamic acid (4 mg/kg IM, preop); T3 = Tolfenamic acid only (4 mg/kg IM, preop). The values are expressed as the means ± SDs or medians [IQRs] depending on the data distribution.

*
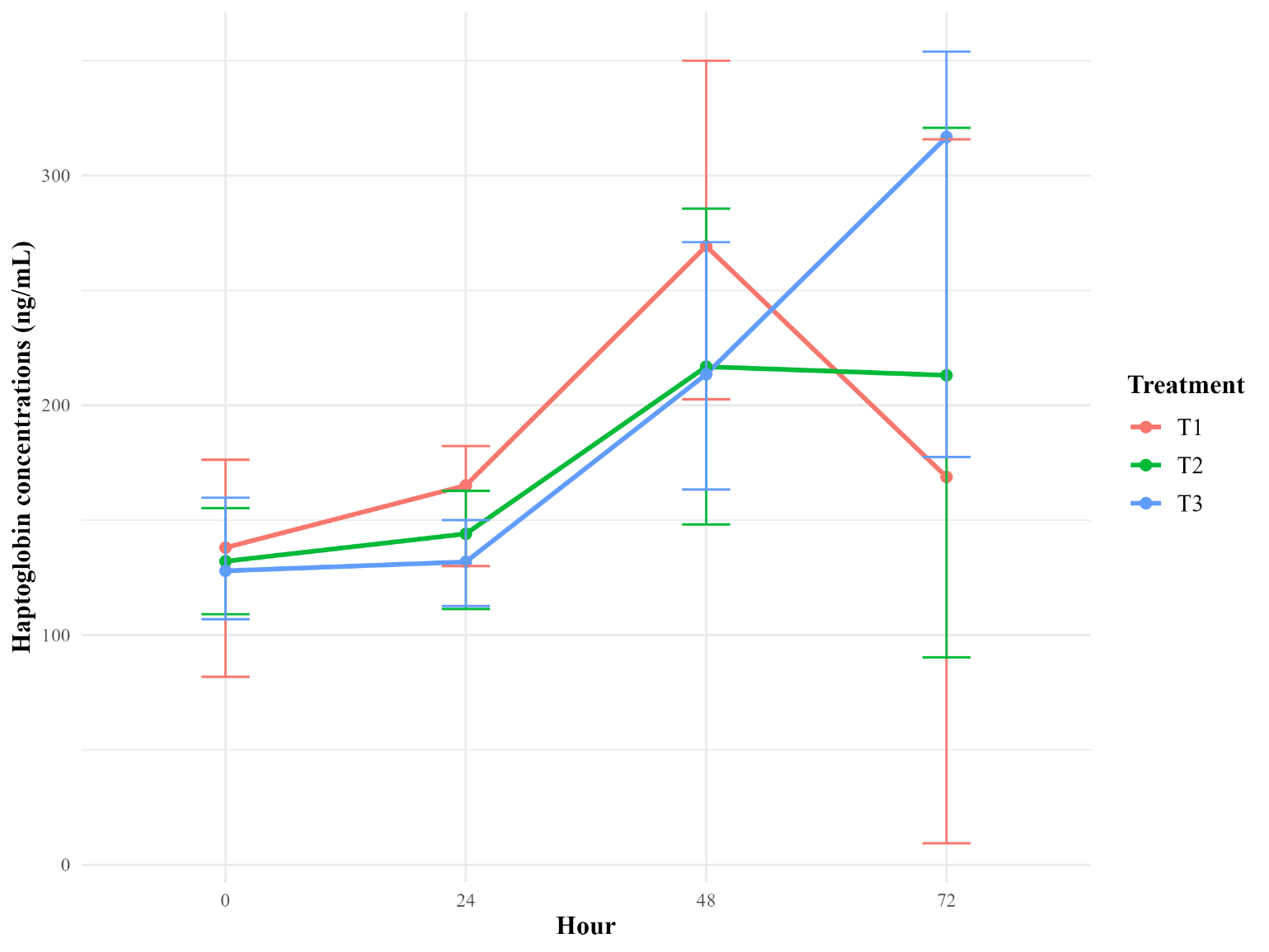
*

**Figure.** Median serum haptoglobin concentrations (ng/mL) in beef calves cast under three analgesic protocols in tropical Colombia at 0, 24, 48, and 72 h postcastration.

T1 = Spermatic cord block with lidocaine (10 mL, 2% per cord) + tolfenamic acid (4 mg/kg IM, preop); T2 = Low epidural anesthesia with lidocaine (4 mL, 2%) + tolfenamic acid (4 mg/kg IM, preop); T3 = Tolfenamic acid only (4 mg/kg IM, preop).


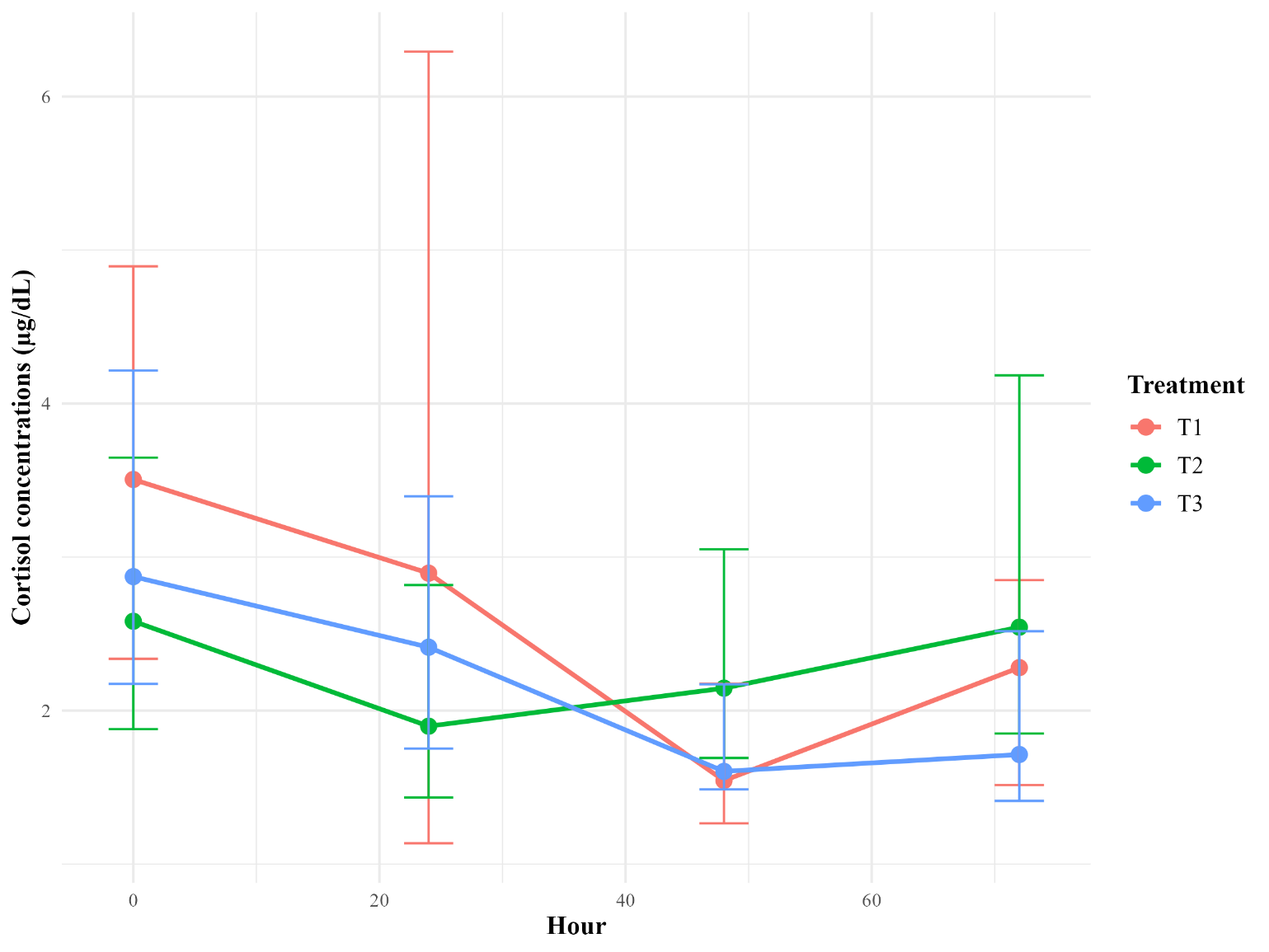


**Figure.** Distribution of serum cortisol concentrations (µg/dL) at different time points after castration (0, 24, 48, and 72 h) in beef calves under three analgesic protocols in tropical Colombia.

T1 = Spermatic cord block with lidocaine (10 mL, 2% per cord) + tolfenamic acid (4 mg/kg IM, preop); T2 = Low epidural anesthesia with lidocaine (4 mL, 2%) + tolfenamic acid (4 mg/kg IM, preop); T3 = Tolfenamic acid only (4 mg/kg IM, preop). The data are shown as boxplots indicating the median (horizontal line), interquartile range (box), and range (whiskers).

**Table.** Temporal evolution of scrotal surface temperature (°C) in beef calves castrated under three analgesic protocols in tropical Colombia.

| **Time (h)** | **n** | **Median** | **Q1** | **Q3** | **IQR** |
| --- | --- | --- | --- | --- | --- |
| 24 | 42 | 32.0 | 30.00 | 33.27 | 3.27 |
| 48 | 42 | 33.7 | 32.73 | 34.38 | 1.65 |
| 72 | 42 | 33.5 | 31.88 | 34.90 | 3.02 |

The values are expressed as medians, first quartiles (Q1), third quartiles (Q3), and interquartile ranges (IQRs).


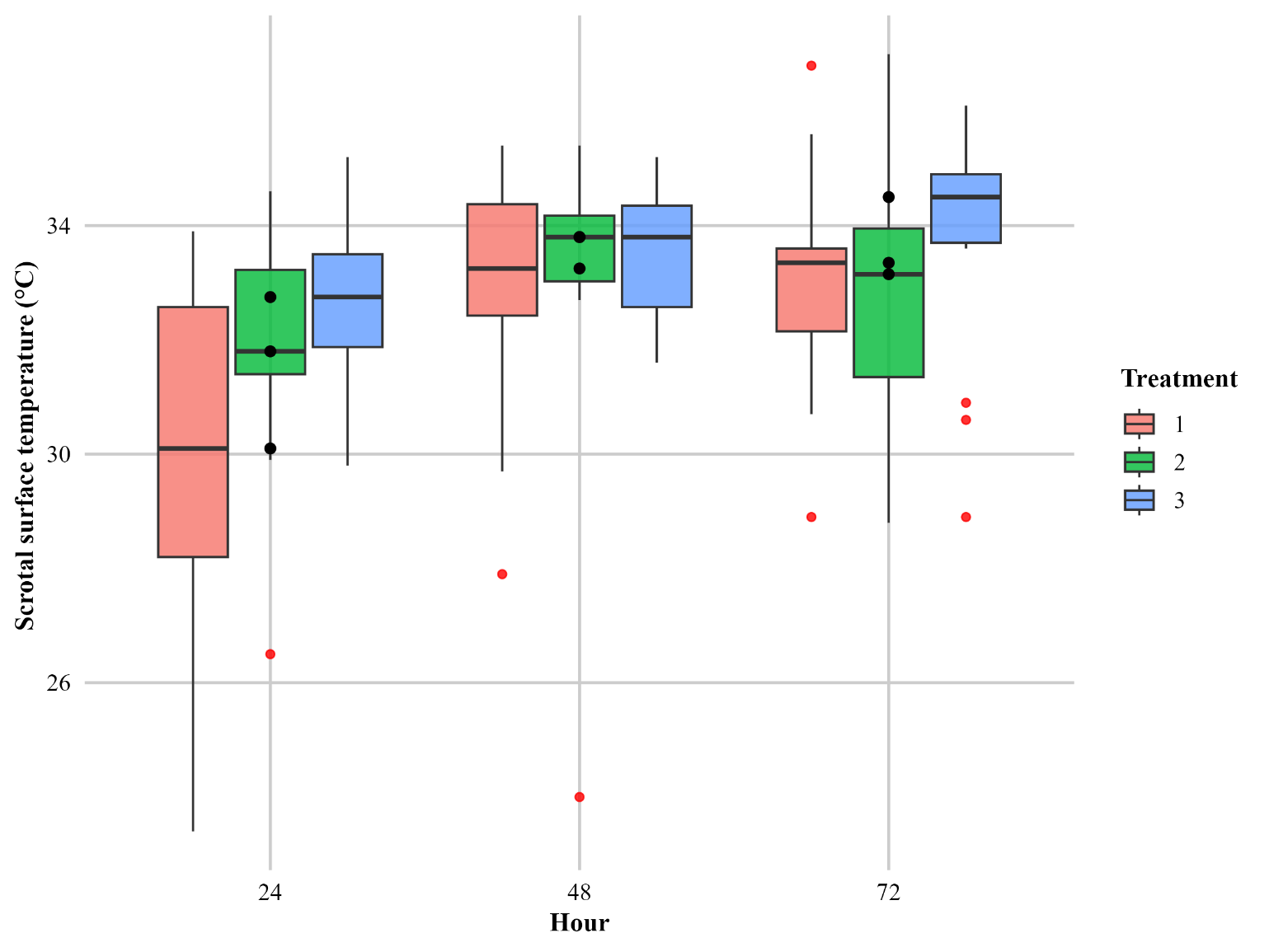


**Figure.** Temporal changes in measured by infrared thermography (IRT) in beef calves under three analgesic protocols in tropical Colombia at 24, 48, and 72 h postcastration.

T1 = Spermatic cord block with lidocaine (10 mL, 2% per cord) + tolfenamic acid (4 mg/kg IM, preop); T2 = Low epidural anesthesia with lidocaine (4 mL, 2%) + tolfenamic acid (4 mg/kg IM, preop); T3 = Tolfenamic acid only (4 mg/kg IM, preop).


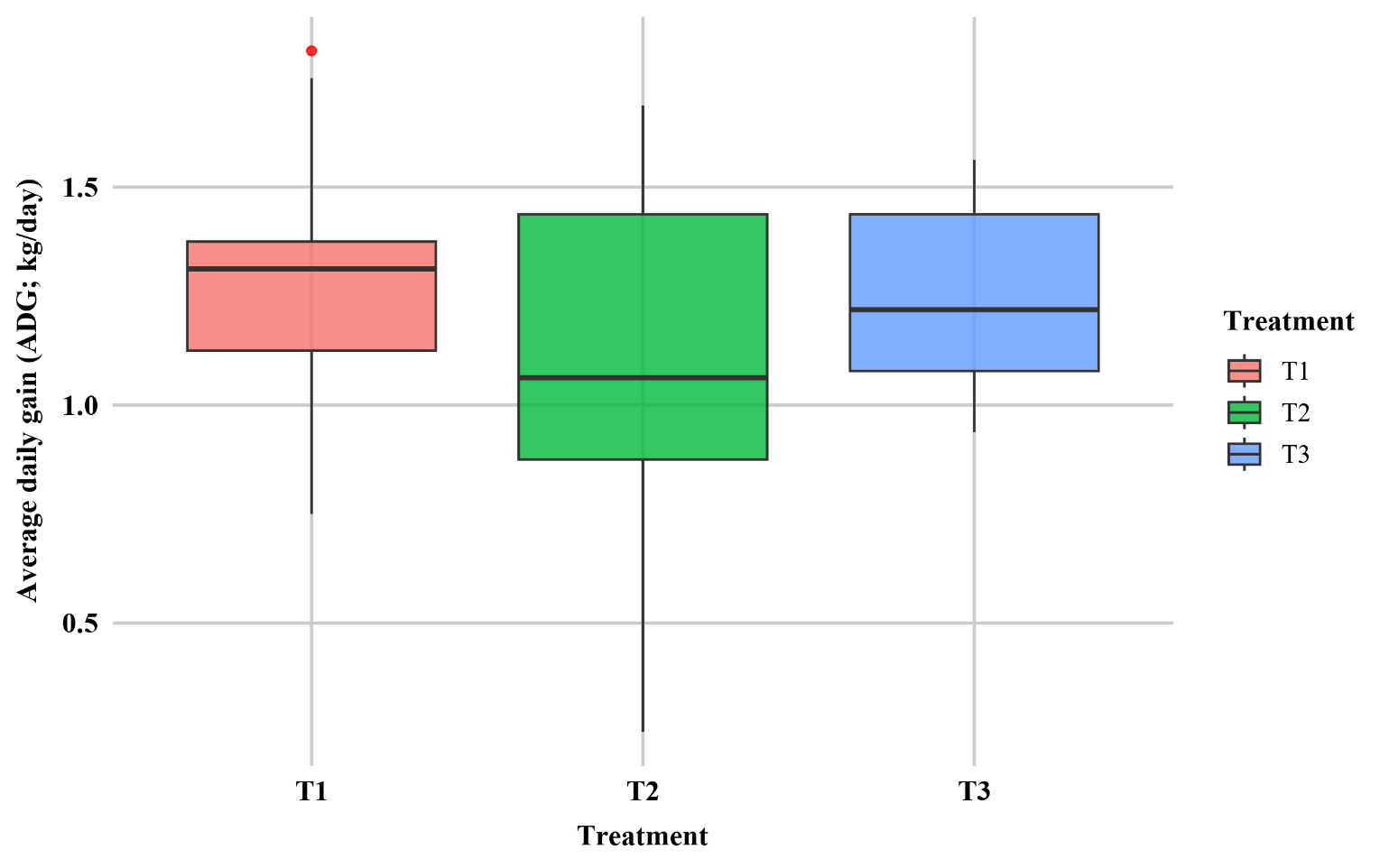


**Figure.** Distribution of average daily gain (ADG; kg/day) in beef calves cast under three analgesic protocols in tropical Colombia.

T1 = Spermatic cord block with lidocaine (10 mL, 2% per cord) + tolfenamic acid (4 mg/kg IM, preop); T2 = Low epidural anesthesia with lidocaine (4 mL, 2%) + tolfenamic acid (4 mg/kg IM, preop); T3 = Tolfenamic acid only (4 mg/kg IM, preop). Boxplots represent the median, interquartile range, and outliers.

**Table.** Summary statistics of average daily gain (kg/day) in beef calves cast under three analgesic protocols in tropical Colombia.

| **Treatment** | **Description** | **n** | **Mean (kg/day)** | **SD** | **Median** | **Min** | **Max** |
| --- | --- | --- | --- | --- | --- | --- | --- |
| T1 | Spermatic cord block with lidocaine (10 mL, 2% per cord) + tolfenamic acid (4 mg/kg IM, preop) | 13 | 1.31 | 0.31 | 1.31 | 0.75 | 1.81 |
| T2 | Low epidural anesthesia with lidocaine (4 mL, 2%) + tolfenamic acid (4 mg/kg IM, preop) | 13 | 1.11 | 0.40 | 1.06 | 0.25 | 1.69 |
| T3 | Tolfenamic acid only (4 mg/kg IM, preop) | 14 | 1.25 | 0.20 | 1.22 | 0.94 | 1.56 |

The values are expressed as the means ± standard deviations (SDs), medians, minimums, and maximums.
